# Hospital Housekeeping

**Published:** 1887-01-29

**Authors:** 


### HOSPITAL HOUSEKEEPING.

It is a recognised principle that necessity's sharp pinch often produces excellent results. For the last few years the financial difficulties of the Newcastle Infirmary have pressed upon its managers with increasing force. As a consequence, a special Committee of Governors was appointed to inquire into the management, and to suggest remedies for the removal of the evils which were sapping the life-blood of this important institution. It has often been declared with confidence that hospitals in the provinces are more energetically and better managed than those in the metropolis. The work performed by the Special Committee of the Newcastle Infirmary will go a long way to confirm such contentions. Its significance at this time, when every hospital in the kingdom is more or less oppressed by financial difficulties, is immense. Besides, the work has been so well done as to make the document, which applies only to one provincial infirmary, of importance and interest to every hospital in the country. Feeling this to be so, we have decided to reproduce it next week in a special supplement, and to offer three prizes to the superintendents, secretaries, governors, or others, who shall write the best report of their hospital, covering the same ground and dealing with all the questions reported upon by the Newcastle Infirmary Committee.

In London and elsewhere it has been too much the fashion to demand an ever-increasing income, and to hold optimistic views concerning expenditure. Hospital housekeeping, and the expenditure under various heads in the different departments of such an institution, have found few critics, and no workers of sufficient energy to undertake a thorough, careful, and exhaustive investigation like that which has just been concluded at Newcastle. We have in preparation a series of papers on the cost of hospital living in London and the provinces, which cannot fail to prove of interest and value. Meanwhile it is interesting to note that the cost per occupied bed for food at Newcastle is £23 3*s.* 10*d.*; at Sheffield it is £20 12*s.* 4*d.*; and at Dundee, £13 17*s.* 3*d.* A difference of forty per cent. per bed between Newcastle and Dundee in the expenditure upon food must at once rivet the attention of thoughtful persons. How is such a difference possible, and what items account for it? Beef costs only £5 9*s.* 4*d.* per occupied bed in Dundee, whilst £10 3*s.* 2½*d.* is paid at Newcastle for this item. Fish costs £1 8*s.* 5¼*d.* in Newcastle, and 16*s.* 5½*d.* in Dundee; bread and flour cost £2 6*s.* 2*d.* at Newcastle, and £1 17*s.* 3*d.* at Dundee; and milk, one of the most important articles of consumption in a hospital, costs £4 14*s.* 3*d.* per occupied bed at Newcastle, and only £2 3*s.* 1½*d.* at Dundee. Were we to take a representative London hospital, as we hope to demonstrate on another occasion, the difference between it and the expenditure at the Newcastle Infirmary would show as great an excess, as we have proved, to exist between the expenditure there and that at the Dundee Infirmary. In the result the Committee are of opinion that, by a careful revision of the dietary by the medical board, the expenditure of food can be reduced below that of Leeds, with advantage to the patients. Such a reduction in the expenditure of the Newcastle Infirmary would be equal to £4 per bed on a total of 235 beds constantly occupied, that is, to a saving of £938 per annum in provisions alone. Attention is further called to the cost per bed occupied at the various provincial hospitals, which varies from a minimum of £42 3*s.* 9*d.* at Sunderland to a maximum of £65 16*s.* 4*d.* at Hull. The cost per occupied bed at the Glasgow Western Infirmary is £50 18*s.*; at the Royal Infirmary, Edinburgh, £56 5*s.*; and at the Birmingham General Hospital, £56 6*s.* These figures are something like 75 per cent. less than the cost of an occupied bed in several of the principal metropolitan hospitals.

Of course, the question of alcohol cropped up, with the result that stimulants were found to cost £1 7*s.* 5*d.* per head at Newcastle, a sum higher than that at any other hospital quoted, except Sheffield, where it amounts to £1 18*s.* 10*d.* per head, although the Sunderland Infirmary spends only 4*s.* 6¾*d.* per head upon alcohol. It ought to be added that the number of persons other than patients included in the above calculations amounted to 85 at Newcastle, 64 at Sheffield, and 44 at Sunderland. One of the most interesting features of the report, and one which is calculated to give rise to much discussion, and to be followed by an alteration of practice in many hospitals, is the statement of Mr. T. Hatfield Walker, F.C.S., analyst for the City of Carlisle, with reference to a sample of beef-tea submitted to him for examination. The committee found that the expenditure upon beef-tea was very large, and Mr. Walker contends that the amount of beef used to a pint, viz., one pound, might be reduced with advantage to ten ounces, provided the meat was cooked at a lower temperature and for a longer time. If this contention be correct, then a saving of £220 would be effected in the expenditure on beef-tea alone, to provide one year's consumption for the occupants of 235 beds. Here then is a question which it would repay every hospital committee and medical officer to thoroughly test and examine.

There are very many other points of interest and instruction, including the non-admission of children to general hospitals, the opening of special wards for paying patients, an alteration in the system of book keeping to show the expenditure on the patients in dressings, drugs, etc., by each member of the medical staff separately, the non-attendance of casualty cases, the limitation of the number of the out-patients, and the payment of a poll-tax of five guineas per head per annum by all medical students. Each and all these questions are of more or less importance, but many of them present novel features, well calculated to give rise to considerable differences of opinion. In any case, the great merit of this report will be generally recognised; and if it lead to an examination of the expenditure by the different committees of all the hospitals in London and throughout the country, it is certain to result, at a moderate estimate, in the saving of something like a quarter of a million of money per annum in the aggregate. Such a result would be worthy of the Jubilee Year; and we would urge the managers of all well-conducted hospitals to bring this question of the expenditure before the annual meetings of governors now being held throughout the country, and to recommend the appointment of a special committee to inquire into all the questions raised by the report of the Newcastle Infirmary.

